# Multicentre RCT and economic evaluation of a psychological intervention together with a leaflet to reduce risk behaviour amongst men who have sex with men (MSM) prescribed post-exposure prophylaxis for HIV following sexual exposure (PEPSE): A protocol

**DOI:** 10.1186/1471-2334-12-70

**Published:** 2012-03-22

**Authors:** Carrie Llewellyn, Charles Abraham, Alec Miners, Helen Smith, Alex Pollard, Paul Benn, Martin Fisher

**Affiliations:** 1 Brighton & Sussex Medical School, Brighton, UK; 2 Peninsula College of Medicine & Dentistry, Exeter, UK; 3 London School of Hygiene & Tropical Medicine, London, UK; 4 Camden Provider Services at Central and North West London NHS Foundation Trust, London, UK; 5 Brighton and Sussex University Hospitals NHS Trust, Brighton, UK

**Keywords:** Sexual behaviour, HIV, Motivational interviewing, Post-exposure-prophylaxis, Intervention

## Abstract

**Background:**

Post-exposure prophylaxis (PEP) following sexual exposure to HIV has been recommended as a method of preventing HIV infection in the UK. Men who have sex with men (MSM) are the group most affected by HIV in the UK and their sexual risk taking behaviour is reported to be increasing. One-to-one behavioural interventions, such as motivational interviewing (MI) have been recommended to reduce HIV in high risk groups. The Information, Motivation and Behavioral skills (IMB) model has been shown to provide a good basis for understanding and predicting HIV-relevant health behaviour and health behaviour change, however the IMB has yet to be applied to PEP after risky sexual exposure. The primary aim of this trial is to examine the impact of MI augmented with information provision and behavioural skills building (informed by the IMB Model), over and above usual care, on risky sexual behaviour in MSM prescribed PEP after potential sexual exposure. A secondary aim of this research is to examine the impact of the intervention on adherence to PEP. This study will also provide estimates of the cost-effectiveness of the intervention.

**Methods:**

A manualised parallel group randomised controlled trial with economic evaluation will be conducted. The primary outcome is the proportion of risky sexual practices. Secondary outcomes include: i) Levels of adherence to PEP treatment; ii) Number of subsequent courses of PEP; iii) Levels of motivation to avoid risky sexual behaviours; iv) Levels of HIV risk-reduction information/knowledge; v) Levels of risk reduction behavioural skills; vi) Diagnosis of anal gonorrhoea, Chlamydia and/or HIV. 250 participants will be asked to self-complete a questionnaire at four time points during the study (at 0,3,6,12 months). The intervention will consist of a two-session, fixed duration, telephone administered augmented MI intervention based on the IMB model. A newly developed treatment manual will guide the selection of persuasive communication strategies as appropriate for each participant and will be based on underlying change mechanisms specified by the IMB theoretical framework. Information provision and skills building will also be included in the intervention package through the use of information leaflets and tailored action plans. Fidelity of intervention delivery will be assessed.

**Discussion:**

The results from this NIHR funded study will identify whether it is appropriate and cost-effective to intervene using one-to-one telephone calls with MSM seeking PEP. If the intervention is effective, further work will be needed on training staff to deliver the intervention competently.

**Trial registration numbers:**

UKCRN ID:11436; ISRCTN00746242.

## Background

The prevalence of HIV infections continue to rise in the UK, particularly amongst MSM and the reduction of new infections is a key component of the National Strategy for Sexual Health and HIV [1]. Recent reports on sexual health highlight the need to invest more in HIV prevention strategies [2,3]. The Community HIV and AIDS prevention (CHAPS) policy (led by the Terrence Higgins Trust) identifies post-exposure prophylaxis following sexual exposure (PEPSE) to the human immunodeficiency virus (HIV) as one method of preventing HIV infection in the UK [4]. Recommendations for treatment are derived from the existing use of antiretrovirals to prevent HIV infection after high risk occupational exposure to the virus ('needle-stick injuries') [5]. Recommendations for prescribing PEP result from the clinician's assessment of risk of transmission. If the risk of HIV transmission through particular sexual practices (such as unprotected anal intercourse) is of a similar magnitude as occupational exposures then PEP should be recommended [6].

Evidence suggests that PEP may reduce the risk of HIV infection if given within 72 hours and adhered to rigorously for 28 days [5,7,8]. Sustained adherence is required to prevent treatment failure, however, non-adherence of a quarter to a third of those prescribed PEP has been reported [9,10]. The treatment can be challenging to patients due to side-effects such as diarrhoea, nausea, headaches and vomiting. The treatment is also costly.

Men who have sex with men (MSM) are the group most affected by the HIV epidemic in the UK [11] and their sexual risk taking behaviour is reported to be increasing [12,13]. As part of a comprehensive strategy across HIV prevention and care, behavioural interventions remain an important tool in the global fight against HIV [14]. One-to-one behavioural interventions, such as motivational interviewing (MI) have been recommended [15,16] to reduce HIV in high risk groups. NICE guidance places recommendations for one-to-one interventions within the context of current STI/HIV service provision [1,17] and states that these interventions are integral to the modernisation of sexual health services [16]. Seeking PEP after potential sexual exposure may indicate an unmet prevention need and provides an opportunity to target interventions thus potentially lowering the likelihood of further risk behaviour [18,19].

We will implement a telephone-administered intervention based on motivational interviewing (MI) augmented with information and behavioural skills building (informed by the Information-Motivation-Behavioral Skills Model). MI is defined as a 'directive, client-centred counselling style for eliciting behaviour change by helping clients to explore and resolve ambivalence' [20]. It is may be especially effective for individuals who are reluctant to change or who are ambivalent about changing their behaviour and may be particularly appropriate for MSM who may require a more tailored preventive strategy. A systematic review and meta-analysis of RCTs shows that MI outperforms traditional advice giving in the treatment of a range of behavioural problems and diseases [21]. More recently, a pilot study has shown the effectiveness of telephone administered MI to reduce risky sexual behaviour in HIV infected rural populations [22].

**Figure 1:**
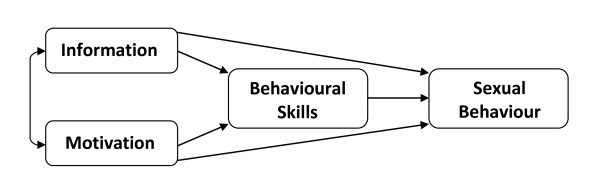
**The Information-Motivation-Behavioral Skills Model (Fisher and Fisher, 1992)**.

The study will examine whether a two-session, telephone administered augmented motivational interviewing intervention based on the IMB model (Figure 1) reduces risky sexual behaviour (compared with 'treatment as usual'), in MSM prescribed PEP treatment after potential sexual exposure to HIV. Evidence suggests that the Information-Motivation-Behavioral Skills (IMB) approach could be used to explain sexual risk taking behaviour [23]. Moreover, the IMB model has been shown to provide a good basis for understanding and predicting HIV-relevant health behaviour and health behaviour change in almost two decades of research [24]. This model further proposes that information relevant to the personal practice of preventive behaviour, motivation to practice prevention and behavioural skills for practicing prevention effectively, are fundamental determinants of HIV/STI preventive behaviour [25]. The IMB model proposes an intervention development process involving (1) elicitation of client resources and needs and, (2) matching intervention content to existing need and behaviour change objectives, (3) implementation of information, motivation and skill development techniques interventions according to protocol and, finally, (4) evaluation.

Interventions based on this approach has been found to be effective in changing risky sexual behaviour among HIV negative and infected individuals in more than 25 studies [26] and in changing levels of adherence behaviour amongst people with HIV/AIDS [27,28]. The IMB has yet to be applied to a short term prophylactic regimen of medication after risky sexual exposure but its previous successful application to HIV risk reduction suggests that it is an appropriate model on which to base an intervention for those prescribed PEP. This study will also provide estimates of the cost-effectiveness of the intervention through the inclusion of an economic evaluation.

## Objectives and hypotheses

The primary aim is to examine the impact of motivational interviewing augmented with information and behavioural skills building (informed by the Information-Motivation-Behavioral Skills Model), over and above usual care, on risky sexual behaviour in MSM prescribed PEP after potential sexual exposure. A secondary aim of this research is to examine the impact of the intervention on adherence to PEP.

Specifically we hypothesize that compared with treatment as usual, those in the intervention arm will:

1) Report a reduction in the proportion of risky sexual practices (less unprotected anal intercourse (UAI) (receptive and insertive), increased use of condoms, a reduction in partners);

2) Have greater levels of adherence to PEP treatment;

3) Have lower rates of subsequent requests for PEP;

4) Have lower incidences of HIV.

5) Have lower incidences of anal gonorrhoea and Chlamydia;

6) Have greater motivation to avoid risky sexual behaviours;

7) Have greater knowledge of risk reduction strategies;

8) Have greater risk reduction behavioural skills.

## Methods

### Study design

**Figure 2:**
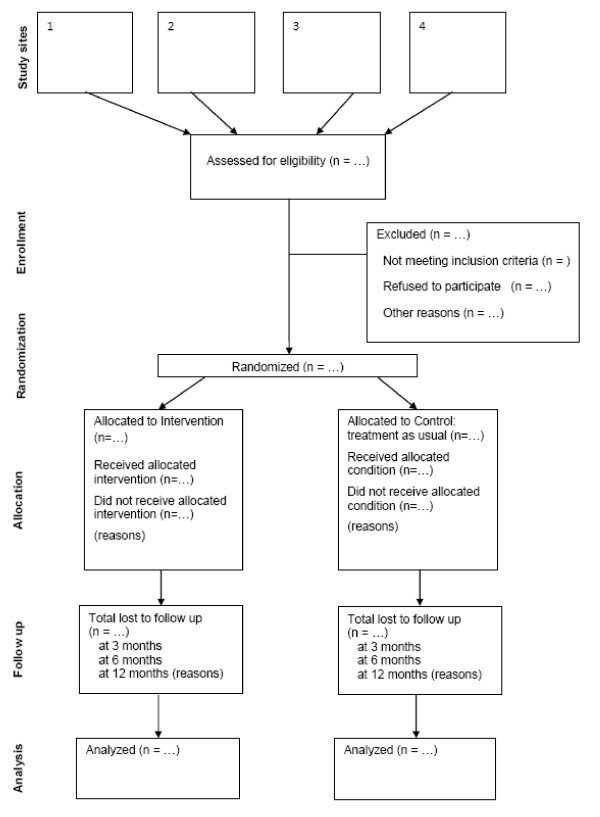
**CONSORT flowchart of study participants**.

Parallel group randomised controlled trial (Figure 2).

### Participants

#### Eligibility criteria

Participants will be MSM, aged ≥16 years, prescribed PEP after sexual exposure, attending a Genito-urinary Medicine (GUM) clinic that are willing and able to give written, informed consent.

#### Exclusion criteria

The following groups of patients will be excluded: people who have received previous psychological support from a clinical psychologist in relation to their sexual risk taking; people with learning difficulties; or those unable to read study materials; or with no means of communication acceptable to the patient; or who are seeking PEP after sexual assault.

### Recruitment procedure

The data collection plan is tailored to the standard patient care protocols stipulated in the BASHH guidelines for PEP [4], and the clinical processes in use at the recruitment sites. PEP is available from GUM or Accident & Emergency (A&E) Departments. Patients are routinely prescribed a 5-day course of PEP medication at general GUM clinics, or at A&E departments outside of GUM hours. Whichever mode of access, all patients attend a dedicated PEP clinic at GUM within 5 days of initial prescription. Recruiting at the GUM clinic will ensure all patients who are prescribed the full course of PEP (28 days) are included in the trial from either access route. Eligible participants will be identified by a research nurse/health advisor at the GUM clinic and given study information and consent forms. Participants have a two-week time period in which to consent and return baseline measures. This two week period allows the recruitment of patients at an early stage in their prescription, but at a time when their immediate anxiety should be reduced and they are able to give considered, informed consent.

### Baseline and follow-up assessments

Participants enrolled into the study will be asked to self-complete a questionnaire at four time points during the study (at 0,3,6,12 months). The baseline questionnaire will be administered at the recruitment stage. Upon receiving the consent forms and baseline assessments, participants will be randomly allocated to either intervention or control conditions (see randomisation section). The second questionnaire will be either posted to patients, accessed via an email link or the internet (as per consent) prior to their 3-month follow-up appointment with the clinic. Any patients who have not returned completed questionnaires before their '3-month' follow-up will be sent repeat copies^1 ^(as per consent), and subsequently called on the telephone (if consented), and finally, may be approached by study staff at their clinic appointment. The third and fourth questionnaires will be posted to patients or accessed via an email link or the internet 6 and 12 months after the end of PEP. Repeat copies, and subsequent telephone calls/text messages, will be used as reminders.

The recruitment period is for 12 months to allow sufficient numbers of participants to be recruited. A sample size of 250 (allowing for a 75% recruitment rate and 50% retention rate) is achievable within the time-frame from four sites and we anticipate that numbers will be higher given the increasing trends for PEP requests.

### Randomization and blinding

Participants will be randomised after consent and collection of baseline measures. The recruiting staff will not have access to the results of randomisation prior to recruiting the participant. Randomisation will occur within each clinic. The allocation sequence will be generated and released to the interventionist on a case-by-case basis by an independent company who specialise in supplying random generated sequences for research. The randomisation sequence will be recorded. This is a single-blind trial, given the nature of the behavioural intervention, blinding of participants is not feasible and the interventionist will know that all those contacted are in the intervention arm. The statistician will be blind to individual results during the trial and the allocation-to-trial-arm coding will be revealed when the dataset is sealed. The interventionist and supervisors will be blind to the baseline and follow-up measures which will not be involved in the delivery of the intervention. Anonymised responses will be entered onto the database by a person unconnected to the project.

## Trial treatment arms

### Control group: Treatment as usual

Both of the groups will receive 'treatment as usual': Patients are initially seen by a HA/SpN for an initial consultation, 5 day prescription of PEP and blood tests (for HIV, Hepatitis B and liver function). Patients receive their first follow-up appointment 5 days later to receive further PEP (if HIV-ve). After the 28 day treatment regimen patients receive either a face-to-face or telephone appointment with the HA/SpN to discuss their sexual health, adherence to PEP and the blood test results. At 4 months after exposure (3 months after the end of PEP) patients are recalled by the HA for HIV testing. In order to assess the impact of the intervention on outcome measures and IMB constructs, those allocated to the control group will be asked to complete all measures at 0,3,6, and 12 months.

### Intervention group

#### Procedure

The intervention group will receive 'treatment as usual' plus the addition of an intervention which will be delivered as two telephone sessions employing motivational interviewing (MI) augmented with information and skills building based on the IMB model of behaviour change. The first telephone call will be made within one week of the participant being consented into the trial and after baseline assessments are received. The second call will occur 7 days (+/-2 days) later. The intervention will be completed by the end of the 28 day course of PEP. A telephone format is proposed to allow the same person to conduct all interventional sessions to control for provider differences and to facilitate recruitment from a wide geographical area in an economical manner. If shown to be effective, this intervention would be sustainable as an improvement to the routine care model.

### Duration and content of intervention

#### Telephone delivered MI

Each telephone session will be a maximum of 30 minutes long. The second session will contain similar content to the first but will reiterate and build on the risk reduction motivation from session 1. In the case of drop-out between the 2 intervention sessions, the dose-response will be assessed.

The interventionist will initially assess individual risk behaviours and any informational, motivational or skill deficits which have contributed to maintenance of participants' risky sexual behaviours and discuss particular areas related to risky sex e.g. the use of alcohol or drugs during sex. The interventionist will elicit self-motivational statements from the participant with the use of open-ended questions and will utilise MI strategies to increase motivation to change, including:

1) Providing the participant with feedback about his risky sexual behaviours

2) Increasing the participant's sense of responsibility to reduce risky sexual behaviours

3) Providing brief and direct advice to create a desire for change

4) Providing a menu of options from which the participant can choose to reduce risk

5) Demonstrating empathy by listening carefully, and accurately understanding his problems

6) Enhancing self-efficacy to reduce risky sexual behaviours [29]

If an individual discontinues with PEP before the end of the treatment, they will still be eligible to continue in the study. Data on adherence to PEP will be captured by the measures of adherence and a review of the medical notes.

#### The role of the manual

The manual will guide the selection of persuasive communication strategies appropriate for each participant and will be based on underlying change mechanisms specified by the IMB theoretical framework. The manual describes how to elicit information and includes scripts describing likely sexual-risk scenarios. Specific behaviour change techniques are identified [30,31] and possible responses exemplified.

#### Information provision and skills building

Information about HIV risk behaviour, prevalence and strategies to minimise risk will be provided to those allocated into the intervention arm ('Ready for Action' Second Edition, and 'Get it on' condom guide both produced by the Terrence Higgins Trust). This will be either sent by post, email or accessed via the internet (as preferred) after baseline measures have been returned but before the intervention. The interventionist will prompt the participant to read the information if they have not already done so.

The skills building component of the intervention will be given during the telephone session in the form of interventionist-provided suggestions of practical strategies needed to master these skills. A well-specified action plan will be developed and agreed on (between interventionist and participant) during the session and the participant will be asked to write this down and enact it. The interventionist will also write down the agreed action plan and send this to the participant after the session (either by post,or email as indicated by participant). Questions about adherence to practicing the action plan are included in the follow-up questionnaires.

#### Treatment fidelity

Assessing the fidelity of the treatment is an important component of successful research dissemination. Translating effective behavioural change interventions from this research setting to clinical practice can be facilitated better when treatment fidelity strategies are used as guidelines for implementing new interventions in clinical contexts. In order to monitor the reliability and validity of the intervention, assessment of both the interventionist and the participant will conducted as per National Institutes of Health (NIH) Behaviour Change Consortium 'best practice' recommendations. Ensuring same treatment dose within conditions will be ensured by a fixed number of sessions and by delivery within the intervention period. Ensuring interventionist skill acquisition and minimising 'drift' in interventionist skills will be minimised by the development and use of a treatment manual; by monitoring and providing feedback to the interventionist; and by providing adequate training. The gold standard method to ensure standard delivery of the intervention is to evaluate or code the sessions (through audiotape) according to predefined criteria. A validated instrument, the Motivational Interviewing Skill Code (MISC) [29], will be used to provide structured feedback, to monitor and document adherence to MI principles during weekly supervision. The interventionist will also be required to complete a process evaluation checklist (adapted from the NHS Health Trainer Handbook [32]) after each intervention session to remind him to include the appropriate skills and content for each intervention and minimise bias. The advisory board will be used to monitor whether the treatment protocol has been adhered to during the recruitment and intervention period. Reduction of differences within treatments will be ensured by the use of one trained interventionist.

## Measures

### Primary outcome

The proportion of risky sexual practices.

### Risk Behaviour outcome measure

A Risk Behaviour outcome measure has been developed to include items of potential HIV transmission-risk sexual behaviour: number of episodes of unprotected anal intercourse (UAI) (receptive and insertive) over a three month time period with individuals of unknown or HIV positive status, consistency of condom use, number of partners using no protection.

(Unprotected receptive oral sex with an HIV + ve person is associated with minimal HIV transmission risk and thus is not included).

### Secondary outcomes (and method of assessment)

i) Levels of adherence to PEP treatment (Morisky Medication Adherence Scale (MMAS) [33] adapted for use in PEP).

ii) Number of subsequent courses of PEP (self-report and medical record review).

iii) Diagnosis of anal gonorrhoea and Chlamydia.^2^

iv) Diagnosis of HIV.

v) Levels of motivation to avoid risky sexual behaviours ('Measures of Motivation to Perform AIDS Preventive Behavior' questionnaire [34] adapted for use with MSM. This questionnaire provides assessment of attitudes, subjective norms and behavioural intentions in relation to HIV risk-reduction).

vi) Levels of HIV risk-reduction information/knowledge ('Health and Relationships Survey' [34] adapted for use with MSM. Items are summed to form an HIV prevention information scale score).

vii) Levels of risk reduction behavioural skills ('Behavioral Skills Measure' [34]. This consists of two subscales which assess the perceived difficulty of reducing HIV risk behaviour and the perceived effectiveness of methods to reduce risk).

With the exception of adherence, all outcome measures will use a retrospective recall period of 'the past 3 months'. Other factors assessed by self-report questionnaire at each time point will be alcohol and substance use. Socio-demographic (age, ethnicity, education, employment, relationship status) will be assessed at baseline. Experience of treatment side-effects will be assessed using a previously used measure at 3 month together with the measure of adherence.

### Frequency and duration of follow-up

The primary and secondary outcomes ii, v, vi, and vii will be collected at 3, 6 and 12 months after the end of treatment (and the end of the intervention). Secondary outcome i. will be collected at 3 months only. Outcomes iii. and iv. will be collected at 12 months only.

The follow-up period is for 12 months to determine intervention sustainability. Participants will be contacted by telephone prior to each follow-up assessment to remind them of their participation in the study and to ask their preference for follow-up (postal, email/internet or face-to-face).

### Analyses plan and power calculation

For the analysis of main effects for the primary outcome a mixed-design ANCOVA will be used with one between-groups factor (treatment vs. control) and one within-groups factor (baseline, 3-,6- and 12-month measures). For this analysis the estimated effect size is f = 0.1 (based on a moderate estimate of effect size from a meta-analysis [24] of one to one interventions to reduce UAI in MSM). 90% power at 0.05 level of significance requires a sample of 250 (125 in each arm). Secondary outcomes will be analysed for the same effect size but with significance adjusted to 0.01 and power to 80%.

### Piloting

All study materials (measures, training manual, intervention, method for data sharing) will be piloted on a sample of MSM and refined in response to their feedback, together with input from the Advisory Board and Steering Group.

### Economic evaluation

The economic evaluation will compare NHS costs of the intervention with usual care. Health outcomes will be expressed as quality-adjusted life-years (QALYs). Decision modelling techniques will be used to extrapolate the RCT results to predict longer term costs and health outcomes. A single economic model with an appropriate time horizon is proposed based on Bernoulli techniques. This approach has been used in a number of previously published studies [35]. This is a conservative approach because it only partially accounts for chains of infections which may be prevented, rather than prevention of single infections.

The purpose of the Bernoulli model is to translate changes in sexual behaviour as observed in the trial into the probability of HIV transmission in order to estimate the number of averted transmissions. To inform this model, baseline data will be collected to allow a more complex infectious diseases model to be built if the intervention is proven. The data are likely to be useful for a range of other HIV modelling studies. Intervention costs will be collected contemporaneously. The longer term costs and health effects of HIV infection will be estimated using published evidence since infection-associated sequelae will not occur during the trial period. Future costs and QALYs will be discounted at 3.5% per annum. Results will be reported as incremental cost per QALYs and cost-effectiveness acceptability curves.

### Vulnerable patients and disclosures

If during the intervention the patient discloses current harmful thoughts or behaviour towards themselves or another, they will be referred to their treating consultant, GP or psychiatric support provided by the GUM clinic. The breaking of confidentiality under these circumstances is detailed on the consent form.

### Anonymity and confidentiality of data

Participants anonymity will be protected by the allocation of a code (not a clinic or NHS number) for use on the database.

### Confidentiality and data sharing between participant sites

All patient-related information to be shared through encrypted NHS email system.

### Ethical approval

The study protocol was approved by the National Research Ethics Service (NRES) Committee South East Coast - Surrey (ref: 11/LO/0718) in 2011.

## Discussion

This paper describes the protocol of a randomised controlled trial to evaluate the impact of motivational interviewing augmented with information and behavioural skills building (informed by the Information-Motivation-Behavioural Skills Model), over and above usual care, on risky sexual behaviour in MSM prescribed PEP after potential sexual exposure. A secondary aim of this research is to examine the impact of the intervention on adherence to PEP. This study will also provide estimates of the cost-effectiveness of the intervention through the inclusion of an economic evaluation.

One of the strengths of this study is that the intervention is targeting individuals who may be deemed as high risk: those who have presented for PEP treatment after a potential sexual exposure. Men who have sex with men (MSM) are the group most affected by the HIV epidemic in the UK [11] and their sexual risk taking behaviour is reported to be increasing [12,13]. As part of a comprehensive strategy across HIV prevention and care, behavioural interventions remain an important tool in the global fight against HIV [14]. One-to-one behavioural interventions, such as motivational interviewing (MI) have been recommended [15,16] to reduce HIV in high risk groups. Although the intervention is tailored to the individual, our proposed intervention is manualised so that the content delivered will be similar and based on the principles of MI with the underlying change mechanisms specified by the IMB Model. As part of this study a manual has been developed. Specific behaviour change techniques used will then be able to be identified and quantified. Translating effective behavioural change interventions from the research setting to clinical practice can be facilitated better when treatment fidelity strategies are used as guidelines for implementing new interventions in clinical contexts. We will be monitoring the reliability and validity of the intervention which is an additional strength of the study.

A recent review [14] highlighted that the recruitment of MSM participants was the most challenging aspect of similar behavioural intervention studies. To overcome these barriers we are employing strategies to ensure that recruitment and retention are maximised, including: the refinement of assessment materials with the MSM community; the use of a telephone-based intervention to overcome geographic boundaries, time constraints and fear of public exposure as an MSM; the recruitment of individuals seeking PEP rather than the recruitment of high risk individuals from the community.

This study will identify whether it is appropriate and cost-effective to intervene using a tailored intervention of one-to-one telephone calls with MSM prescribed the prophylactic treatment regimen after potential sexual exposure to HIV. If the intervention is effective, further work will be needed on training staff to deliver the intervention competently.

## Endnotes

^1 ^Maximum of 2 repeat copies and 1 text/call/email if consented by patient.

^2 ^Incidence data on rectal gonorrhoea and Chlamydia will be collected as this is directly related to the risky sexual behaviour that we are assessing in this trial i.e. directly related to condom use.

## Competing interests

The authors declare that they have no competing interests.

## Authors' contributions

CL, MF, CA, HS, AP, PB, AM contributed to the design of the study and co-authored this article. All authors have read and approved the final manuscript.

## Pre-publication history

The pre-publication history for this paper can be accessed here:

http://www.biomedcentral.com/1471-2334/12/70/prepub
